# A Circulating Proteomic Signature of Allostatic Load Is Associated with Multisystem Disease and Mortality

**DOI:** 10.3390/proteomes14030047

**Published:** 2026-09-10

**Authors:** Yufan Guan, Jie Shen, Li Li, Kai Zhang, Song Liu, Qianqian Zhu, Philip I. Chow, Hongmei Jiang, Jing Li, Lifang Hou, Hua Zhao

**Affiliations:** 1 Department of Public Health Sciences, School of Medicine, University of Virginia, Charlottesville, VA 22903, USA; 2 Department of Family Medicine, School of Medicine, University of Virginia, Charlottesville, VA 22903, USA; 3 Departments of Public Health, University of North Texas Health Science Center at Fort Worth, Fort Worth, TX 76107, USA; 4 Department of Biostatistics and Bioinformatics, Roswell Park Cancer Institute, Buffalo, NY 14263, USA; 5 Department of Psychiatry and Neurobehavioral Sciences, School of Medicine, University of Virginia, Charlottesville, VA 22903, USA; 6 Department of Statistics and Data Science, Northwestern University, Evanston, IL 60208, USA; 7 Division of General Internal Medicine & Population Science, University of Alabama at Birmingham, Birmingham, AL 35233, USA; 8 Department of Preventive Medicine, Feinberg School of Medicine, Northwestern University, Chicago, IL 60611, USA

**Keywords:** allostatic load, proteomes, mortality, morbidity, chronic diseases

## Abstract

**Background:** Chronic stress contributes to the development of cardiometabolic, malignant, and other chronic diseases through cumulative multisystem physiological dysregulation, conceptualized as allostatic load (AL). However, traditional AL relies on heterogeneous clinical biomarkers that are frequently unavailable or inconsistently collected in large cohorts, limiting reproducibility and translational utility. We developed and validated ProAL50, a proteomics-based measure of AL derived from 50 circulating proteins. **Methods:** Using high-dimensional plasma proteomic data from the UK Biobank, we constructed ProAL50 via penalized regression and stability selection and externally validated its construct in the Coronary Artery Risk Development in Young Adults (CARDIA) Study. **Results:** Among 37,089 UK Biobank participants, ProAL50 correlated strongly with traditional AL in the held-out test set (*r* = 0.72) and showed moderate external construct validity in CARDIA (*r* = 0.62). ProAL50 closely mirrored traditional AL in its associations with sociodemographic characteristics, lifestyle behaviors, physical and mental health, inflammation, and biological aging, supporting construct validity. In prospective analyses, ProAL50 showed associations with incident chronic disease, including type 2 diabetes, ischemic heart disease, chronic lung disease, chronic liver disease, chronic kidney disease, overall cancer, and all-cause and cause-specific mortality that were broadly comparable to traditional AL. Representative associations included incident type 2 diabetes (HR per SD = 2.72, 95% CI 2.61–2.84) and chronic liver disease (HR = 2.12, 95% CI 1.96–2.29). **Conclusions:** These findings support ProAL50 as a biologically informative, protein-based surrogate of AL for research use that complements traditional AL, pending further external validation and assay standardization.

## 1. Introduction

Chronic stress is increasingly recognized as a fundamental determinant of population health, influencing the onset and progression of cardiovascular, metabolic, neuropsychiatric, and malignant diseases. The biological imprint of chronic stress is conceptualized through allostasis and allostatic load (AL), a multisystem index of cumulative physiological dysregulation across neuroendocrine, metabolic, inflammatory, and cardiovascular pathways [1,2]. AL has been widely used in epidemiologic studies and consistently predicts frailty, multimorbidity, functional decline, incident chronic disease, and mortality [3,4,5,6].

Despite its conceptual significance, the practical application of traditional AL has been constrained by several methodological and translational limitations. Traditional AL indices rely on clinical laboratory biomarkers such as blood pressure, lipid profiles, glucose metabolism, inflammatory markers, and anthropometric measures. A fundamental barrier is that many large cohort studies do not collect laboratory testing at all, leaving the biomarkers needed to construct AL entirely unavailable. Even when laboratory data are collected, there is significant variation in how they are obtained: the panels differ in which biomarkers are measured, samples may or may not require fasting, and assays vary across laboratories and platforms, so the specific variables an AL index demands are often incomplete or inconsistent. As a result, AL frequently cannot be constructed or must rely on incomplete biomarker sets, imputation, or participant exclusion, all of which weaken the resulting estimates. Moreover, there is no standardized algorithm for selecting biomarkers or defining high-risk cut-points; studies differ widely in biomarker inclusion, scoring method, and weighting, undermining reproducibility and comparability [7,8,9]. Together, these limitations impede clinical translation and highlight the need for more biologically grounded and standardized measures.

Advances in high-throughput proteomics offer an opportunity to modernize the measurement of AL. Circulating proteins integrate genetic, environmental, behavioral, and contextual influences and capture dynamic, system-wide physiological states. Large-scale proteomic platforms such as Olink Explore panels provide standardized, reproducible quantification of thousands of plasma proteins with minimal sample requirements. Emerging work demonstrates that proteomic signatures can robustly index biological aging, systemic inflammation, immune activation, and chronic disease risk [10,11,12,13]. For example, proteomics-based aging clocks outperform many clinical biomarkers in predicting morbidity and mortality [10,12]. These features make proteomics particularly well suited for capturing the multisystem dysregulation that underlies chronic stress. However, it should be noted that affinity-based proteomic platforms primarily quantify protein abundance rather than resolving individual proteoforms, which may provide additional biological information relevant to chronic stress physiology.

We hypothesized that a parsimonious plasma proteomic signature trained to predict traditional AL could capture multisystem physiological dysregulation through a circulating proteomic signature, complementing (not replacing) traditional AL measurement and providing a practical option for studies where individual AL biomarkers are not consistently available. Using UK Biobank Pharma Proteomics Project data (N = 53,013 with Olink Explore measurement of 2922 proteins), we developed ProAL50, a 50-protein signature trained to predict AL, and externally validated the construct in the Coronary Artery Risk Development in Young Adults (CARDIA) cohort (N = 2796). We benchmark ProAL50 against traditional AL; test its associations with incident chronic disease, cause-specific mortality, and patient-reported health outcomes; and characterize the biological pathways it represents. ProAL50 is a supervised signature anchored to traditional AL, so it inherits both the strengths of the AL construct (integrated multi-system measurement) and some of its limitations. Its contribution is to provide a parsimonious, continuous, and interpretable proteomic representation of AL biology, measurable using a standardized research proteomic platform of chronic stress and its related chronic disease, when individual biomarkers are unavailable.

## 2. Methods

### 2.1. Study Participants

We analyzed data from the UK Biobank, a large population-based cohort of more than 500,000 adults aged 40–69 years enrolled between 2006 and 2010. Participants with available proteomic measurements, AL components, and complete covariate and outcome information were eligible for inclusion. Proteomic profiling was available for 53,013 participants through the UK Biobank Pharma Proteomics Project (UKB-PPP), which included a large, broadly sampled subset together with smaller subsets selected through consortium-specific disease-focused projects [14]. Olink ((Olink Proteomics AB, Uppsala, Sweden) proteomic data were provided as Normalized Protein Expression (NPX) values on a log2 scale, after centralized preprocessing and quality control performed by the UK Biobank. Detailed information on sample selection, laboratory processing, and quality control procedures is available in the UK Biobank documentation (https://biobank.ndph.ox.ac.uk/ukb/label.cgi?id=1839, accessed on 24 September 2025). External validation was performed in the Coronary Artery Risk Development in Young Adults (CARDIA) Study, a prospective, community-based cohort that enrolled 5115 Black and White adults aged 18–30 years in 1985–1986 from four U.S. field centers (Birmingham, AL, USA; Chicago, IL, USA; Minneapolis, MN, USA; and Oakland, CA, USA), with recruitment balanced by race, sex, age, and education. Participants were re-examined periodically thereafter. The present analysis used the Year 25 examination (2010–2011), at which plasma proteomic measurements were available for the external validation sample (N = 2796). In CARDIA, an AL score was reconstructed from component biomarkers comparable to those used in the UK Biobank where available, and ProAL50 was computed by applying the fixed UK Biobank-derived protein weights without model re-estimation.

### 2.2. AL Calculation

We constructed an AL score using eleven physiological biomarkers measured at baseline (Supplementary Table S1), building on the canonical allostatic load framework established by Seeman, McEwen, and colleagues in the MacArthur Studies of Successful Aging [1,2,3,4,5] and following our prior implementations [15,16]. The biomarkers encompassed three major physiological domains: cardiovascular (systolic blood pressure, diastolic blood pressure, and pulse rate), inflammatory (C-reactive protein), and metabolic (high-density lipoprotein cholesterol, low-density lipoprotein cholesterol, waist-to-hip ratio, total cholesterol, triglycerides, hemoglobin A1c, and creatinine). Each biomarker was dichotomized using clinically established high-risk thresholds, with participants assigned a value of 1 for high-risk and 0 otherwise. In addition, individuals reporting use of medications for metabolic disorders or hypertension were assigned a high-risk score of 1. The AL score was calculated as the sum of high-risk indicators (range, 0–12), with higher values indicating greater cumulative physiological dysregulation. Participants without sufficient biomarker data to calculate an AL score were excluded from the analysis (N = 12,139). A total of 40,874 participants were included in the final score construction. After excluding participants with prevalent disease at baseline, 37,089 participants remained in the primary analytic sample. Subsequent analyses were restricted to participants with complete exposure, covariate, and outcome data required for each model, resulting in outcome-specific analytic sample sizes.

### 2.3. Elastic Net Model Development

To identify proteomic predictors of AL, we used Elastic Net regularization with the continuous AL score as the primary training outcome and standardized protein abundance levels as predictors. Protein measurements were preprocessed by the UK Biobank. The Olink platform assayed 2922 proteins, of which 2919 candidate protein variables were available in the model-development dataset. Proteins with excessive missingness were excluded, and remaining missing values were imputed using median imputation. The analytic dataset was randomly divided into a 70% training subset and a 30% held-out validation subset. Protein abundance values were standardized using the means and standard deviations estimated exclusively from the training data, and the same training-derived imputation and scaling parameters were then applied to the held-out test data to avoid information leakage. Elastic Net models were implemented using the glmnet framework. To evaluate the Elastic Net mixing parameter, we examined a prespecified α grid of 0.3, 0.5, 0.7, and 1.0, where α = 1.0 represents the L1-penalized boundary case. For each α value, the regularization parameter λ was selected using 10-fold cross-validation within the training set. Both *λ*_min_ and *λ*_1 − *SE*_ were evaluated, and *λ*_1 − *SE*_ was used for final feature selection to promote parsimony, reduce overfitting, and improve generalizability. The final ProAL model used α = 0.7 because it provided a balance between sparsity, predictive performance, and feature-selection stability while allowing correlated proteomic features to be jointly retained. Sensitivity analyses across the α grid showed that the top-ranked proteins were highly concordant across α values, supporting the robustness of the final α choice. Model performance was assessed in the held-out test set using the correlation between predicted and observed AL.

### 2.4. Protein Feature Selection and Score Construction

Elastic Net regularization was used to identify a parsimonious subset of proteins associated with AL and to construct the ProAL50 proteomic score. The final Elastic Net model was fitted in the training set using continuous AL as the outcome and standardized protein abundance values as predictors. Coefficients were extracted at *λ*_1 − *SE*_. Feature-selection stability was assessed using 1000 resampling iterations within the training data. In each iteration, 80% of the training participants were randomly sampled, and the Elastic Net model was refitted using the same modeling framework. For each resampled dataset, λ was selected using 10-fold cross-validation, and proteins with non-zero coefficients at *λ*_1 − *SE*_ were recorded. Selection frequency was calculated as the proportion of resampling iterations in which each protein was selected and was used to assess feature-selection stability. For construction of the primary ProAL50 panel, proteins with non-zero coefficients in the final Elastic Net model fitted to the full training set were ranked by the absolute magnitude of their coefficients, and the top 50 proteins were retained. For each selected protein, the Elastic Net coefficient was normalized by dividing it by the sum of the absolute values of all coefficients among the 50 selected proteins. Before score calculation, missing protein values were imputed using the protein-specific medians derived from the UK Biobank training set, and each protein was standardized using the corresponding training-set mean and standard deviation. The ProAL50 score for participant k was calculated as:

$$\mathrm{ProAL}50_{k}=\sum_{i=1}^{50}w_{i}z_{\mathrm{ik}}$$

where *z_ik_* is the standardized level of the *i*-th protein for participant *k*, and *w_i_* is the normalized Elastic Net coefficient for that protein (i.e., $w\_i=\frac{\beta_{i}}{\sum_{j=1}^{50}\left|\beta_{j}\right|}$). No intercept was included in the operational ProAL50 score. This weighted score reflects the aggregate weighted influence of the top 50 proteins identified by the Elastic Net model. The score was computed in both the training and test cohorts using identical weights derived exclusively from the training set. Higher ProAL50 values indicate a proteomic profile more strongly associated with higher AL.

### 2.5. Sensitivity Analyses for AL Definition and Protein Panel Size

To evaluate whether the ProAL signature was robust to alternative AL outcome definitions, we performed additional Elastic Net analyses using a three-level AL outcome defined as low, intermediate, and high AL. The resulting three-level AL-trained score was compared with the continuous AL-trained ProAL50 score using correlation analyses and overlap of selected proteins.

To assess whether the choice of a 50-protein panel was arbitrary, we constructed additional panel-size sensitivity scores using the top 20, 100, and 200 stability-ranked proteins, referred to as ProAL20, ProAL100, and ProAL200. These sensitivity scores were constructed using stability-ranked proteins and the same coefficient-weighting approach used in the primary score. We compared ProAL20, ProAL50, ProAL100, and ProAL200 by assessing pairwise score correlations, discrimination of high versus low AL using the area under the receiver operating characteristic curve, and paired DeLong tests comparing alternative panel sizes with ProAL50. Incremental Area Under the Curve (AUC) gain per additional protein was also evaluated to assess diminishing returns across increasing panel sizes. ProAL50 was retained as the primary score because it improved discrimination compared with the sparser ProAL20 panel while retaining most of the signal captured by larger ProAL100 and ProAL200 panels in a more parsimonious and interpretable format. Sensitivity analyses evaluating alternative panel sizes and Elastic Net α values are reported in Supplementary Table S3.

### 2.6. Internal and External Validation

Internal validation was conducted in the held-out UK Biobank test set to evaluate the reproducibility and robustness of ProAL50. The fixed ProAL50 protein list and weights derived from the training set were applied directly to the test set. Associations between ProAL50 and AL were evaluated using Pearson and Spearman correlation analyses. Additional internal validation analyses examined the consistency of ProAL50 across alternative AL scoring approaches, including continuous and categorical AL definitions. External validation was performed in the independent CARDIA Year 25 cohort to assess external construct validity/transportability of the fixed ProAL50 signature. The CARDIA study was conducted using the Olink Explore platform. Protein measurements were normalized using the same procedures as those used in the UK Biobank. Of the 50 proteins included in ProAL50, 48 were available and included in the analysis. The ProAL50 score was generated by applying the fixed UK Biobank-derived protein weights to the corresponding proteins in CARDIA without model re-estimation. In parallel, AL was reconstructed in the external dataset using comparable component definitions and scoring procedures when available. Associations between ProAL50 and externally derived AL were evaluated using Pearson and Spearman correlation coefficients.

### 2.7. Outcome Ascertainment

Incident disease outcomes and cause-specific mortality outcomes were ascertained using linked hospital inpatient, cancer registry, and death registry records. Incident outcomes included overall cancer, site-specific cancers, type 2 diabetes, ischemic heart disease, cerebrovascular disease, emphysema/Chronic obstructive pulmonary disease (COPD), chronic liver disease, chronic kidney disease, Alzheimer’s disease, and Parkinson’s disease and parkinsonism. Site-specific cancers included breast, bowel (colorectal), lung, kidney, prostate, pancreatic, cervical, ovarian, bladder, esophageal, liver, brain, stomach, non-Hodgkin lymphoma, and leukemia. For each incident outcome, participants with the corresponding prevalent disease at baseline had already been excluded. Outcome-specific analytic sample sizes subsequently differed according to the availability of complete exposure, covariate, and outcome data. Outcomes were defined using International Classification of Diseases, 10th Revision (ICD-10) codes, which are listed in Supplementary Table S2. Mortality outcomes were defined according to the underlying cause of death recorded in linked death registry data. Sex-specific cancers were analyzed in the relevant population, including breast, cervical, and ovarian cancers among female participants and prostate cancer among male participants.

### 2.8. Statistical Analysis

Associations of sociodemographic and lifestyle factors with AL and the ProAL50 proteomic score were examined using multivariable linear regression. Each outcome (AL or the ProAL50 score) was modeled separately as a continuous dependent variable. Covariates included age group, sex, race/ethnicity, household income, educational attainment, Townsend deprivation index, smoking status, alcohol consumption, physical activity level, and sleep disturbance category. All predictors were coded as categorical variables, with reference categories specified in Table 1. Associations between indicators of general and mental health with AL and ProAL50 were examined using multivariable linear regression models. To facilitate comparison between AL and ProAL50, both indices were standardized before modeling; therefore, regression coefficients represent mean differences in SD units relative to the reference category for each exposure. Walking pace, self-rated health, fatigue frequency, ProAgeGap category, neutrophil-to-lymphocyte ratio (NLR), anxiety, depression, and anxiety/depression disorders were modeled as categorical variables based on established definitions. All models were adjusted for the same sociodemographic and lifestyle covariates listed above. Associations between AL, ProAL50 score, and incident disease outcomes were assessed using Cox proportional hazards models. AL and ProAL50 were standardized before Cox regression modeling so that hazard ratios for both markers represent the relative hazard per 1-SD increase and are reported on the same standardized scale. Time-to-event was calculated from baseline to incident diagnosis, death, loss to follow-up, or end of registry linkage. Administrative follow-up was censored on 31 December 2022. Outcome-specific numbers at risk, numbers of events or deaths, and median follow-up with interquartile range are reported in Supplementary Tables S5b, S6a, S7b and S8a. For each exposure–outcome pair, we fitted hierarchical models: (1) an unadjusted crude model; (2) Model 1, adjusted for demographic, socioeconomic, lifestyle, and behavioral factors. Hazard ratios (HRs) and 95% confidence intervals (CIs) were reported with corresponding *p* values. Proportional hazards assumptions were evaluated using Schoenfeld residuals, with no significant violations detected. Cox models were also used to examine associations between AL, ProAL50 score, and mortality outcomes. For mortality analyses, crude and Model 1 estimates were presented as the primary association models, and mutually adjusted models containing both AL and ProAL50 were additionally fitted to assess their independent associations. The same analytic framework was applied to examine associations with both the incidence and mortality of site-specific cancers, with each cancer type analyzed separately. To compare predictive discrimination between the two measures, we compared Model 1 plus AL with Model 1 plus ProAL50. The corresponding ΔC was calculated as C(Model 1 + ProAL50) − C(Model 1 + AL). To evaluate incremental predictive information beyond traditional AL, we additionally fitted a mutually adjusted model containing Model 1 covariates, AL, and ProAL50. HRs and 95% CIs for both AL and ProAL50 were estimated from these mutually adjusted models. Incremental ΔC beyond AL was calculated as C(Model 1 + AL + ProAL50) − C(Model 1 + AL). For each outcome, C-indices and ΔC contrasts were recomputed on the same outcome-specific complete-case analytic sample across the compared models, ensuring that differences in discrimination reflected model composition rather than differences in the participants contributing to each C-index estimate. Uncertainty for both ΔC measures was quantified using nonparametric bootstrap resampling with 1000 replicates, and 95% confidence intervals were obtained from the bootstrap percentile distribution. These analyses were performed for incident disease and mortality outcomes. As a sensitivity analysis addressing the possibility that baseline HbA1c and creatinine may reflect subclinical or near-term type 2 diabetes and chronic kidney disease, respectively, we performed 2-year landmark analyses for these two outcomes. Participants who developed the corresponding outcome or were censored within the first 2 years after baseline were excluded, and follow-up was re-indexed from the 2-year landmark. The same multivariable Cox modeling framework used in the primary analyses was then applied. Functional enrichment analysis was performed using STRING (version 12.0; STRING Consortium, Copenhagen, Denmark), with the 50 ProAL50 proteins as the query set and the 2922 Olink-assayed proteins as the reference background. Of these, 2891 were successfully mapped to STRING identifiers and constituted the effective background. Enrichment significance was evaluated using FDR correction. Enrichment results were used to support the biological interpretation of the ProAL50 proteomic signature. All analyses were performed using R (version 4.3.0), with statistical significance defined as two-sided *p* < 0.05. *p* values were corrected for multiple comparisons via the FDR using the Benjamini–Hochberg method.

## 3. Results

### 3.1. ProAL50: A Proteomic Signature of AL

**Figure 1:**
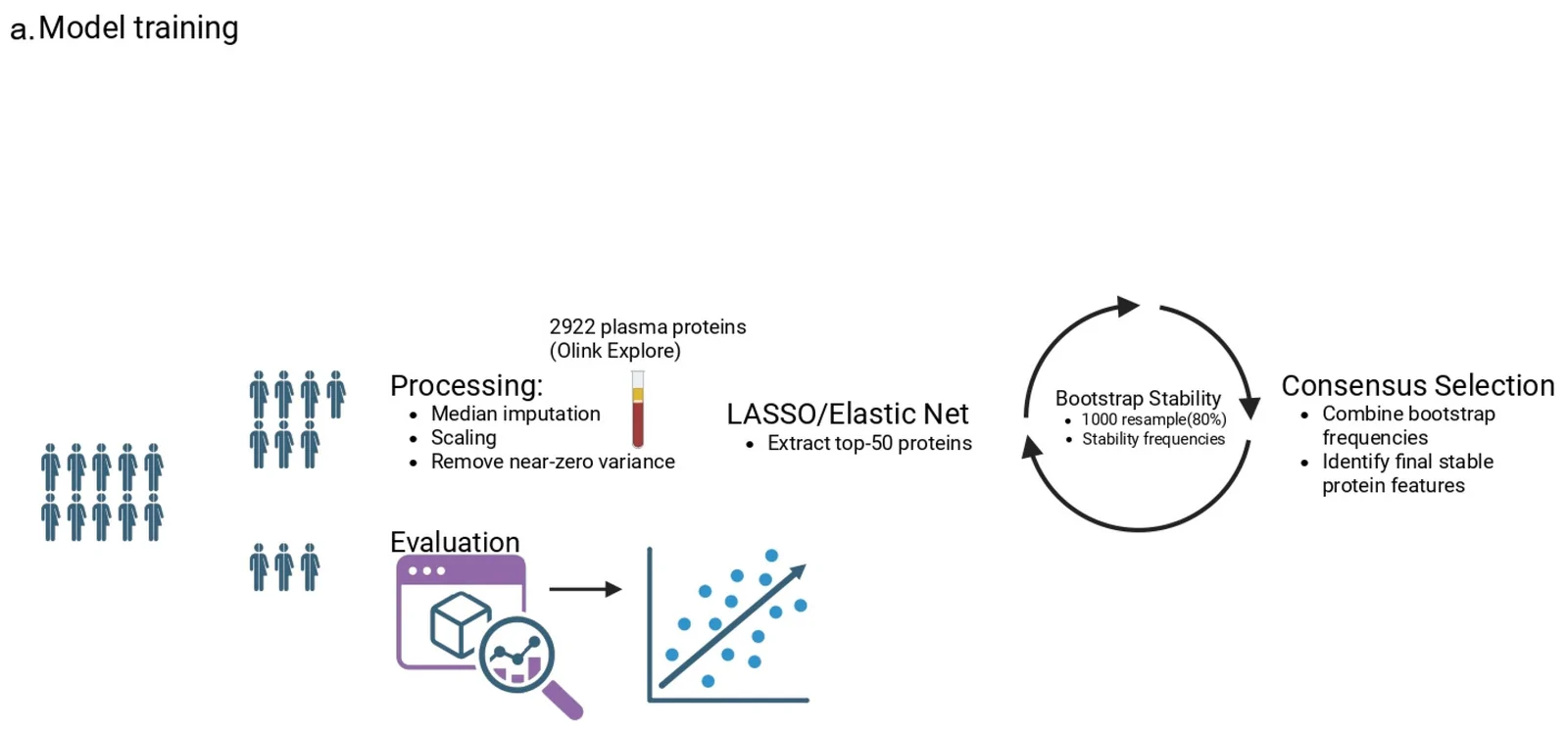

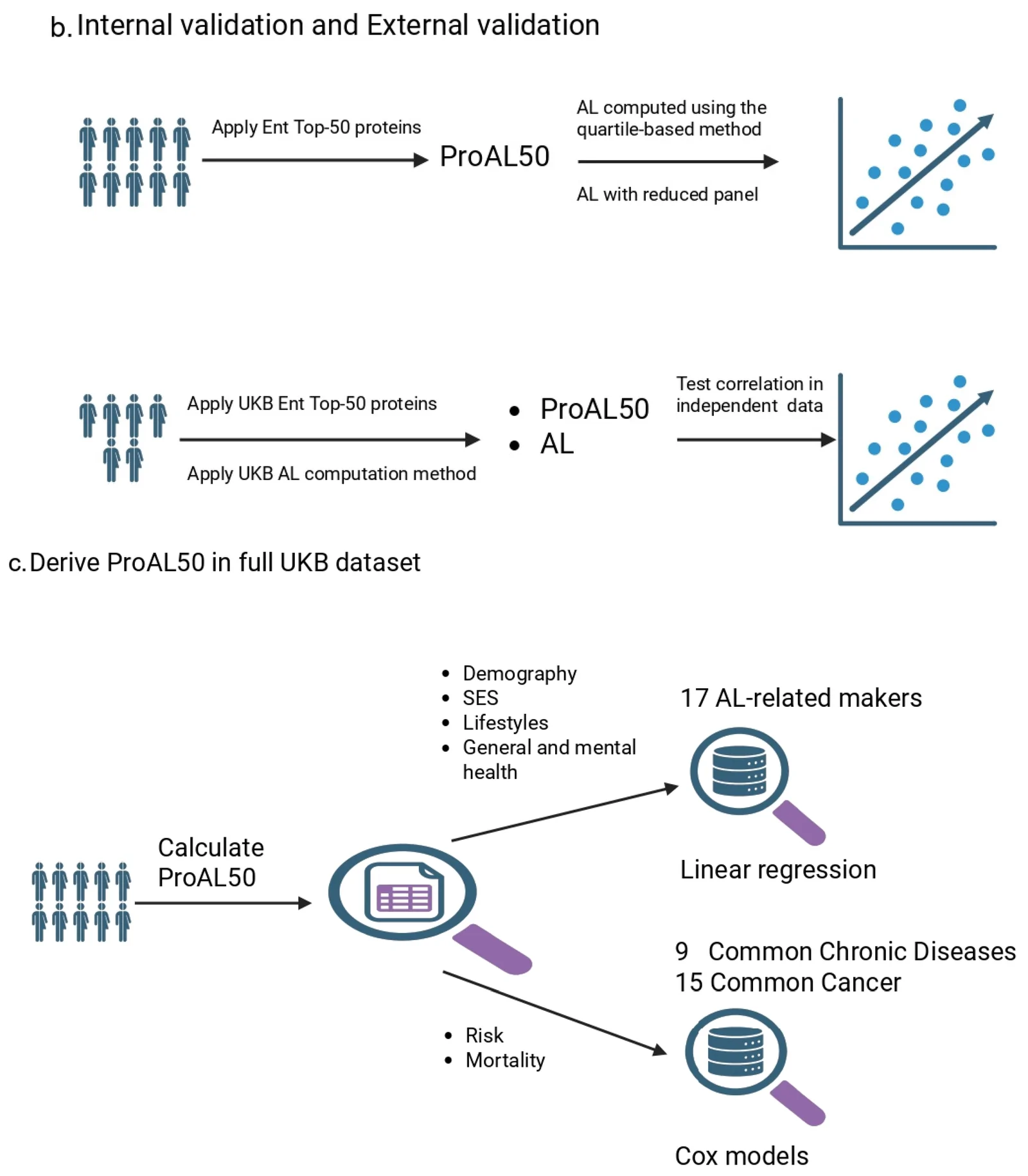
**Development, validation, and application of ProAL50.** (**a**) Derivation of the ProAL50 proteomic score from 2922 plasma proteins using penalized regression and bootstrap stability selection in the UK Biobank. (**b**) Internal validation in the UK Biobank test set and external validation in the CARDIA Study (Year 25) by comparison with established allostatic load measures. (**c**) Application of ProAL50 to sociodemographic, lifestyle, health, disease, cancer, and mortality outcomes using regression and survival analyses.

A schematic representation of the study design and main analytic approaches is shown in Figure 1. The UK Biobank cohort was randomly split into a 70% training set and a 30% internal test set for proteomic score development and validation (Figure 1a). In the training phase, LASSO/elastic net models were fitted to predict traditional AL, and feature-selection stability was evaluated across 1000 resampling iterations, and the final 50 proteins were selected from the fitted training-set Elastic Net model based on the absolute magnitude of their coefficients. Internal validation of ProAL50 was performed within the UK Biobank by comparing the proteomic score with multiple established AL constructs in the independent test set (Figure 1b). External validation was performed using an independent cohort from the CARDIA Study (Year 25). The downstream application of ProAL50 is illustrated in Figure 1c, which outlines the analytic framework used to relate ProAL50 to demographic characteristics, socioeconomic status, lifestyle factors, general and mental health measures, chronic disease burden, cancer incidence, and mortality outcomes.

**Figure 2:**
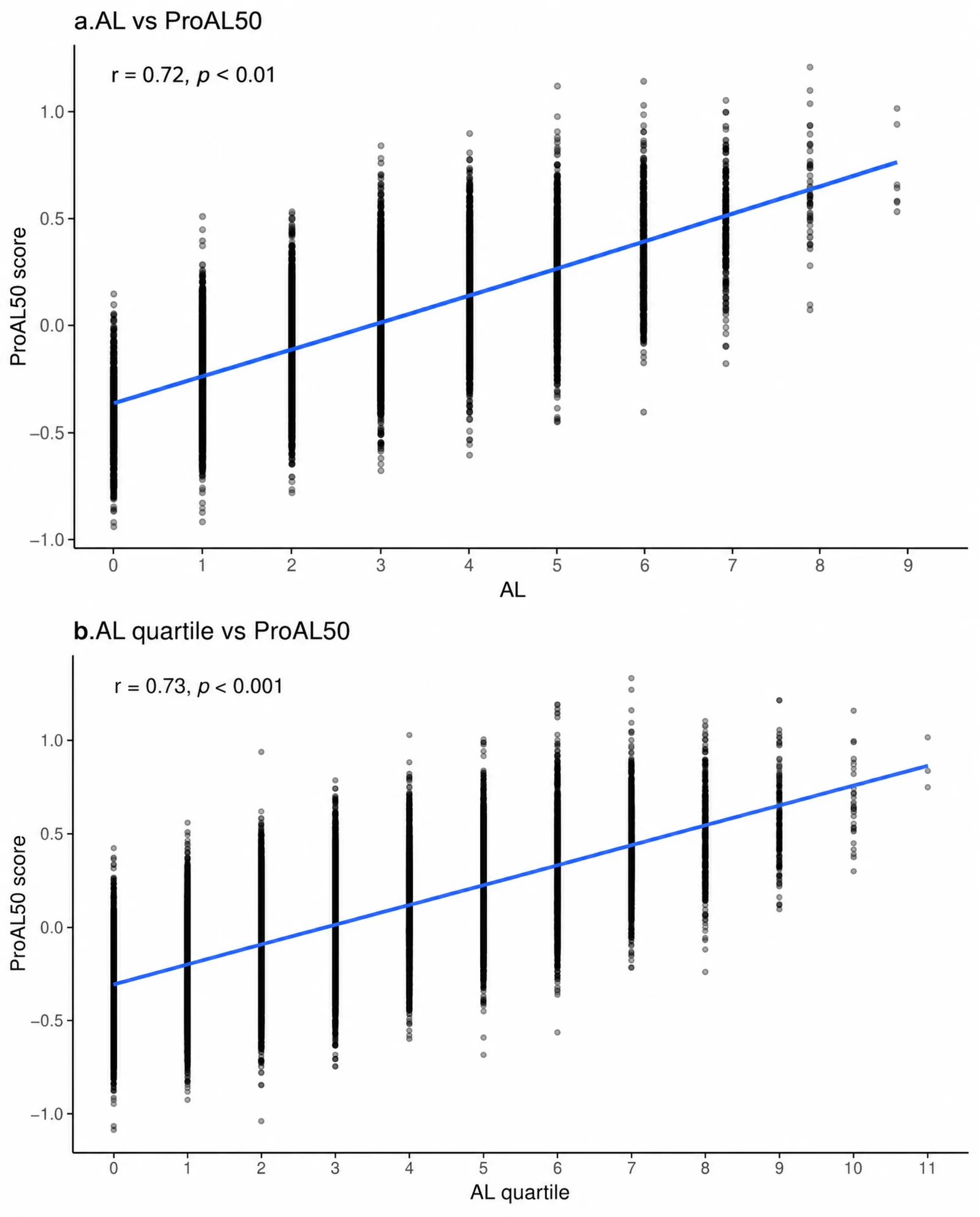

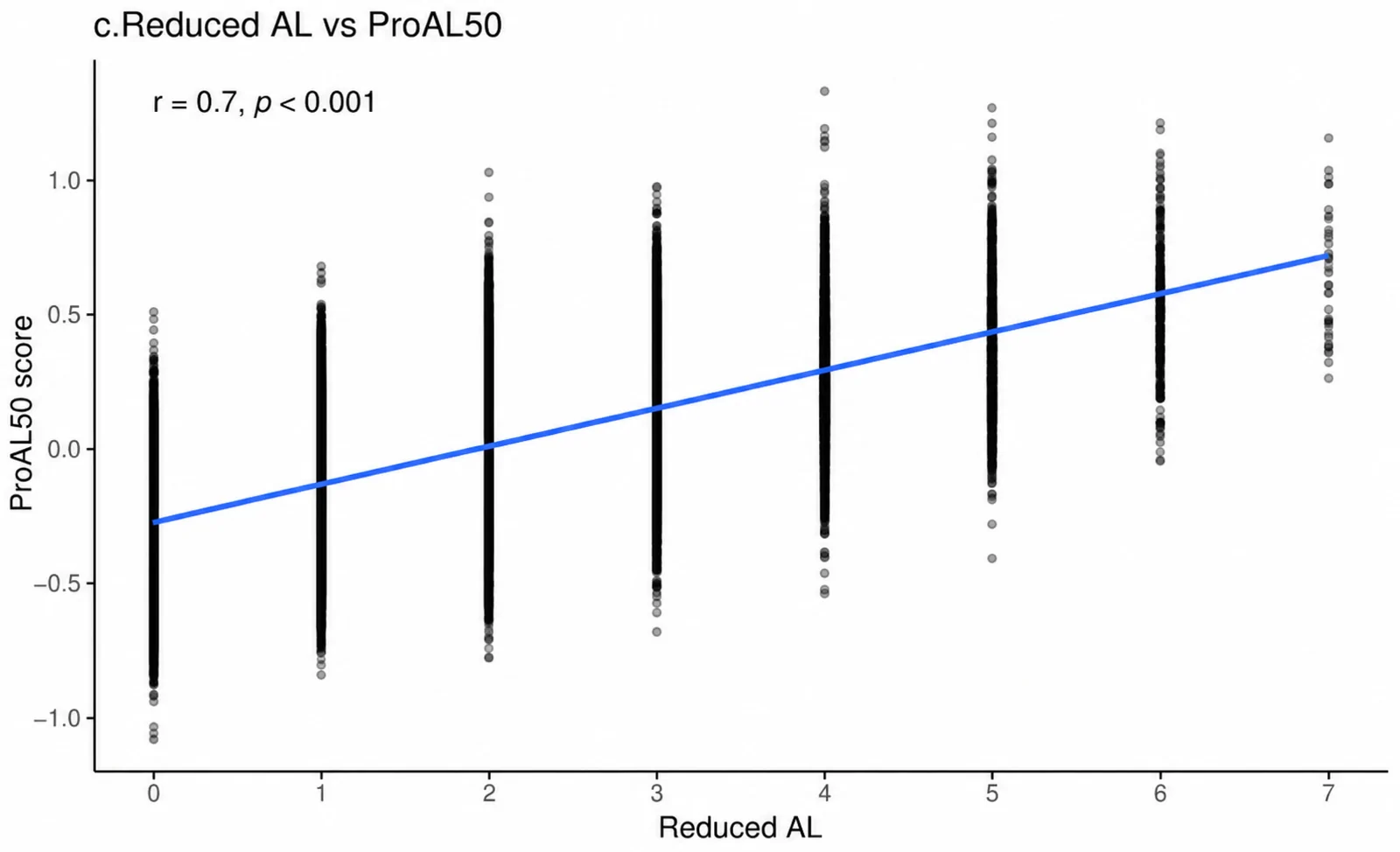

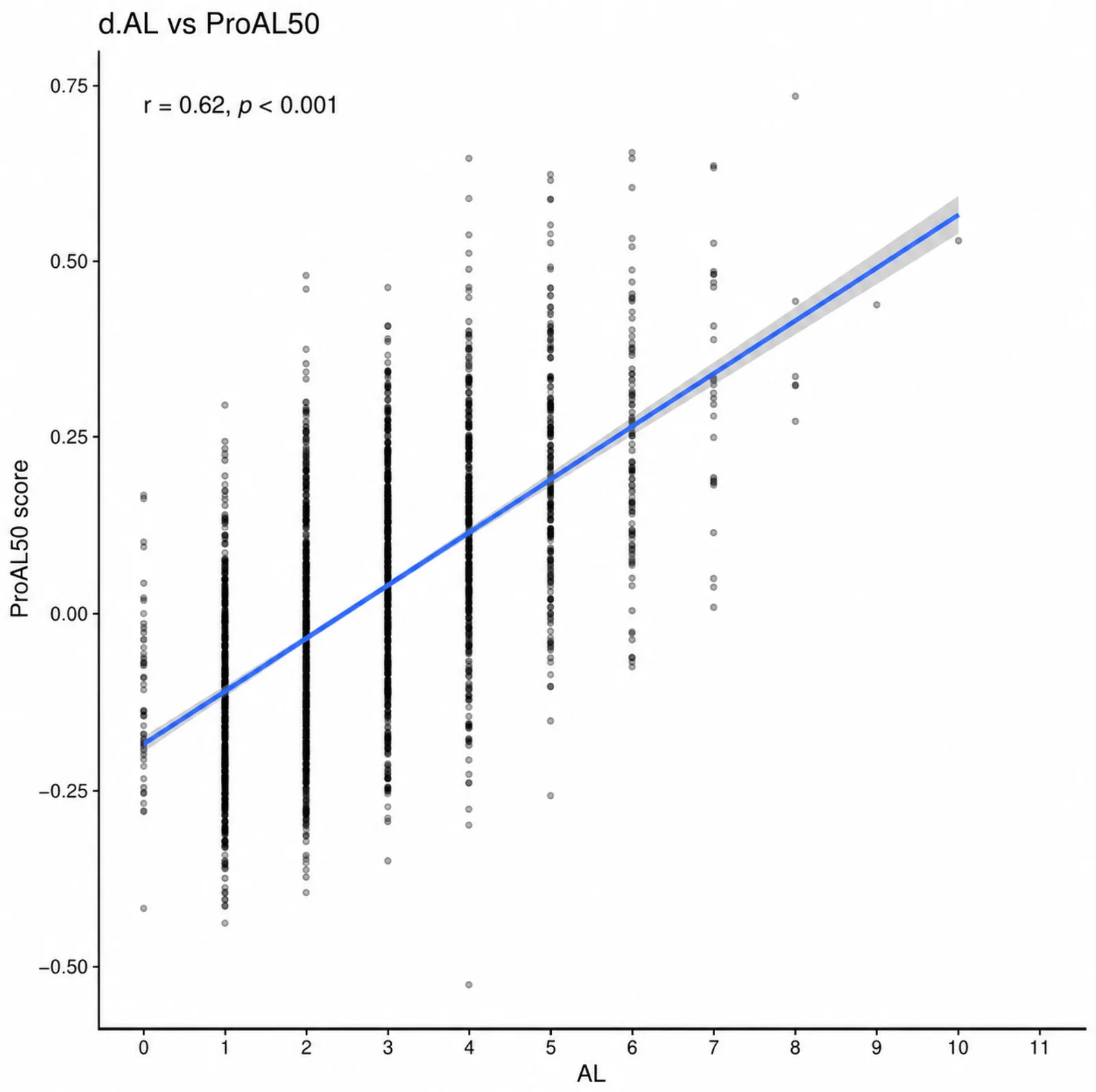
**Correlation between ProAL50 and established allostatic load measures.** (**a**) Correlation between continuous AL and the ProAL50 score in the held-out UK Biobank test set. (**b**) Association between quartile-based AL and ProAL50 in the UK Biobank test set. (**c**) Correlation between ProAL50 and an AL derived from a reduced biomarker panel. (**d**) External validation showing the association between AL and ProAL50 in the CARDIA Study (Year 25). Lines indicate fitted linear regression with 95% confidence intervals; Pearson correlation coefficients (*r*) and *p* values are shown.

Using high-dimensional plasma proteomic data from the Olink Explore platform, we developed ProAL50 using Elastic Net penalized regression, with bootstrap resampling used to assess feature-selection stability. The final 50 proteins were selected from the fitted training-set Elastic Net model based on the absolute magnitude of their coefficients. Model performance was evaluated in the held-out test set by applying the ProAL50 model derived from the training set and correlating the resulting ProAL50 score with observed AL, demonstrating strong agreement (Pearson *r* = 0.72, *p* < 0.001; Figure 2a). Internal validation further supported the robustness of ProAL50, showing high concordance with quartile-based AL (Pearson *r* = 0.73, *p* < 0.001; Figure 2b) and with a reduced AL panel (Pearson *r* = 0.70, *p* < 0.001; Figure 2c). External validation in an independent cohort from the CARDIA Study (Year 25) demonstrated external construct validity (Pearson *r* = 0.62, *p* < 0.001; Figure 2d).

To support the robustness of ProAL50, we conducted sensitivity analyses comparing protein signatures of 200, 100, 50, and 20 proteins (Supplementary Table S3). Although ProAL100 and ProAL200 achieved higher AUCs than ProAL50 (0.881 and 0.890 vs. 0.868, respectively), these differences were statistically significant by paired DeLong tests (*p* = 2.20 × 10^−119^ and *p* = 4.35 × 10^−185^, respectively). The marginal AUC gain per added protein declined progressively, 7.4 × 10^−4^, 2.7 × 10^−4^, and 9.0 × 10^−5^ relative to the 20-protein panel, indicating diminishing returns beyond 50 proteins and supporting our selection of 50 proteins for the signature. ProAL50 was also highly correlated with ProAL20, ProAL100, and ProAL200 (coefficients close to 1.0), indicating that it captured the major AL-related proteomic signal while maintaining a more parsimonious panel. In addition, the top 50 selected proteins were identical across Elastic Net α values of 0.3, 0.5, 0.7, and 1.0, supporting robustness to the choice of the mixing parameter. Finally, ProAL50 was robust to alternative AL outcome definitions: the continuous-AL-derived and three-level-AL-derived ProAL50 scores were highly correlated (Pearson *r* = 0.965, 95% CI 0.964–0.966), and both showed strong correlations with traditional AL.

### 3.2. Sociodemographic and Lifestyle Correlates of ProAL50

In analyses of sociodemographic and lifestyle determinants (Table 1), ProAL50 demonstrated patterns of association highly consistent with those observed for traditional AL. Male showed the strongest demographic association with both measures, followed by older age (>57 years). Racial differences were modest: compared with White participants, Asian participants exhibited higher AL and ProAL50, whereas Black participants showed no difference in AL but lower ProAL50. Indicators of higher socioeconomic status, including higher household income and educational attainment, were consistently associated with lower AL and ProAL50, while greater neighborhood deprivation (higher Townsend index) was associated with higher values of both measures.

Unhealthy lifestyle behaviors were uniformly associated with higher stress-related burden. Both former and current smoking were associated with higher AL and ProAL50 compared with never smoking, whereas alcohol consumption showed a graded inverse association, with moderate and heavy drinkers exhibiting lower values than never drinkers. Physical activity showed the strongest protective association, with moderate and high activity levels associated with substantially lower AL and ProAL50 in a dose–response manner. Sleep disturbance was positively associated with both indices, with progressively higher values observed among participants reporting occasional or frequent sleeplessness.

### 3.3. General and Mental Health Associated with ProAL50

**Figure 3:**
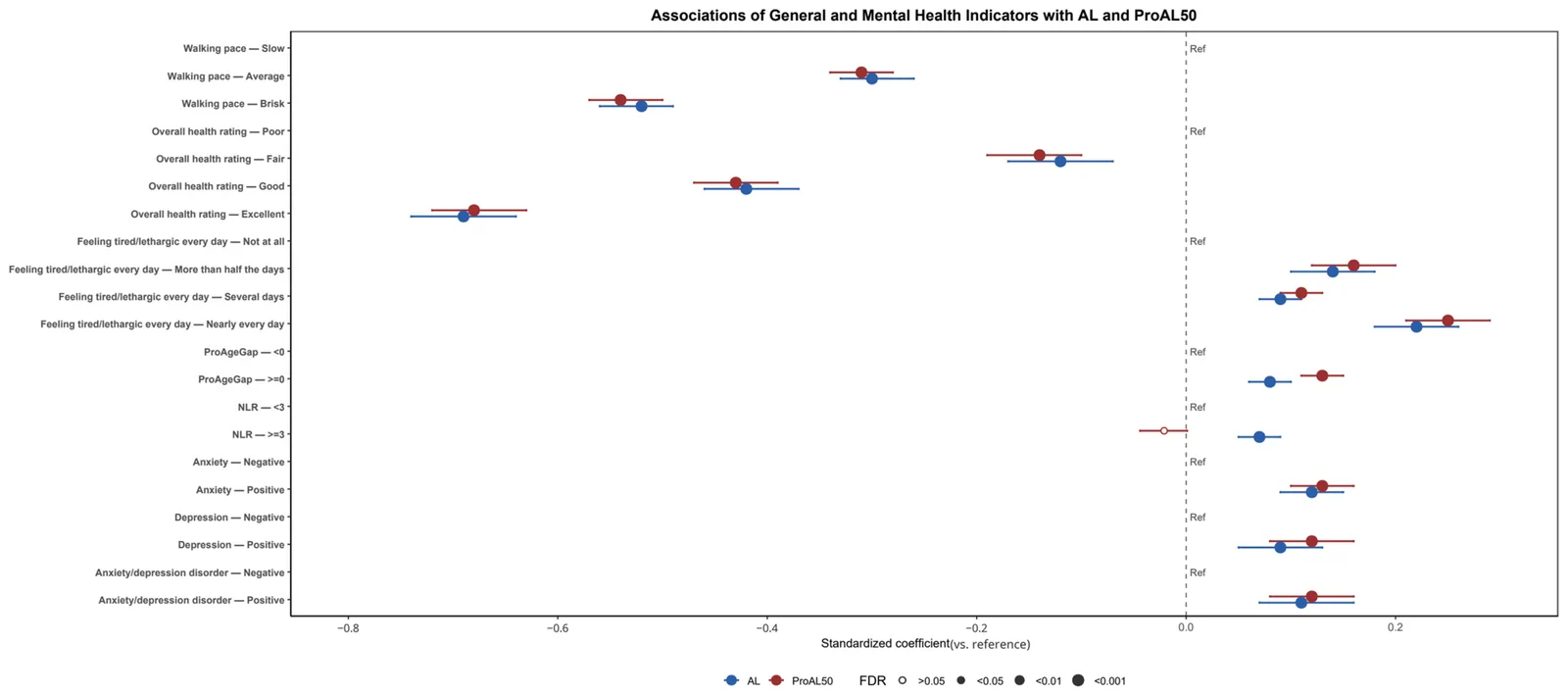
**Associations of general and mental health indicators with allostatic load and ProAL50.** Multivariable linear regression estimates showing associations of general health and mental health indicators with AL and the ProAL50 proteomic score in the UK Biobank. AL and ProAL50 were standardized before modeling; regression coefficients represent mean differences in SD units relative to the reference category. Models were adjusted for demographic, socioeconomic, and lifestyle covariates. Point size denotes false discovery rate (FDR)-adjusted significance levels, and open points indicate non-significant associations (FDR > 0.05).

Measures of general and mental health showed strong, graded, and largely concordant associations with standardized AL and ProAL50 (Figure 3 and Supplementary Table S4). Regression coefficients represent mean differences in SD units relative to the reference category. Better physical functioning and self-rated health were consistently associated with lower AL across indices. Compared with participants reporting a slow walking pace, those reporting a brisk pace exhibited substantially lower AL (β = −0.52, 95% CI −0.56 to −0.49) and ProAL50 (β = −0.54, 95% CI −0.57 to −0.50). Similarly, excellent self-rated health was strongly associated with lower stress-related burden compared with poor health (AL: β = −0.69, 95% CI −0.74 to −0.64; ProAL50: β = −0.68, 95% CI −0.72 to −0.63).

In contrast, markers of poorer health showed parallel positive associations. Greater fatigue frequency was associated with higher AL and ProAL50, with participants reporting near-daily fatigue exhibiting higher AL (β = 0.22, 95% CI 0.18 to 0.26) and ProAL50 (β = 0.25, 95% CI 0.21 to 0.29) compared with those reporting no fatigue. Individuals with a positive ProAgeGap (≥0) also had higher AL (β = 0.08, 95% CI 0.06 to 0.10) and ProAL50 (β = 0.13, 95% CI 0.11 to 0.15). Inflammatory and mental health markers showed similar patterns, with the exception of NLR: elevated NLR (≥3) was associated with higher AL (β = 0.07, 95% CI 0.05 to 0.09), whereas the corresponding association with ProAL50 was small and inverse (β = −0.021, 95% CI −0.044 to 0.001) and did not remain statistically significant after FDR correction (FDR = 0.066). Participants with anxiety had higher AL (β = 0.12, 95% CI 0.09 to 0.15) and ProAL50 (β = 0.13, 95% CI 0.10 to 0.16), with comparable associations observed for depression and comorbid anxiety/depression.

### 3.4. ProAL50 Predicts Risk of Common Chronic Diseases

**Figure 4:**
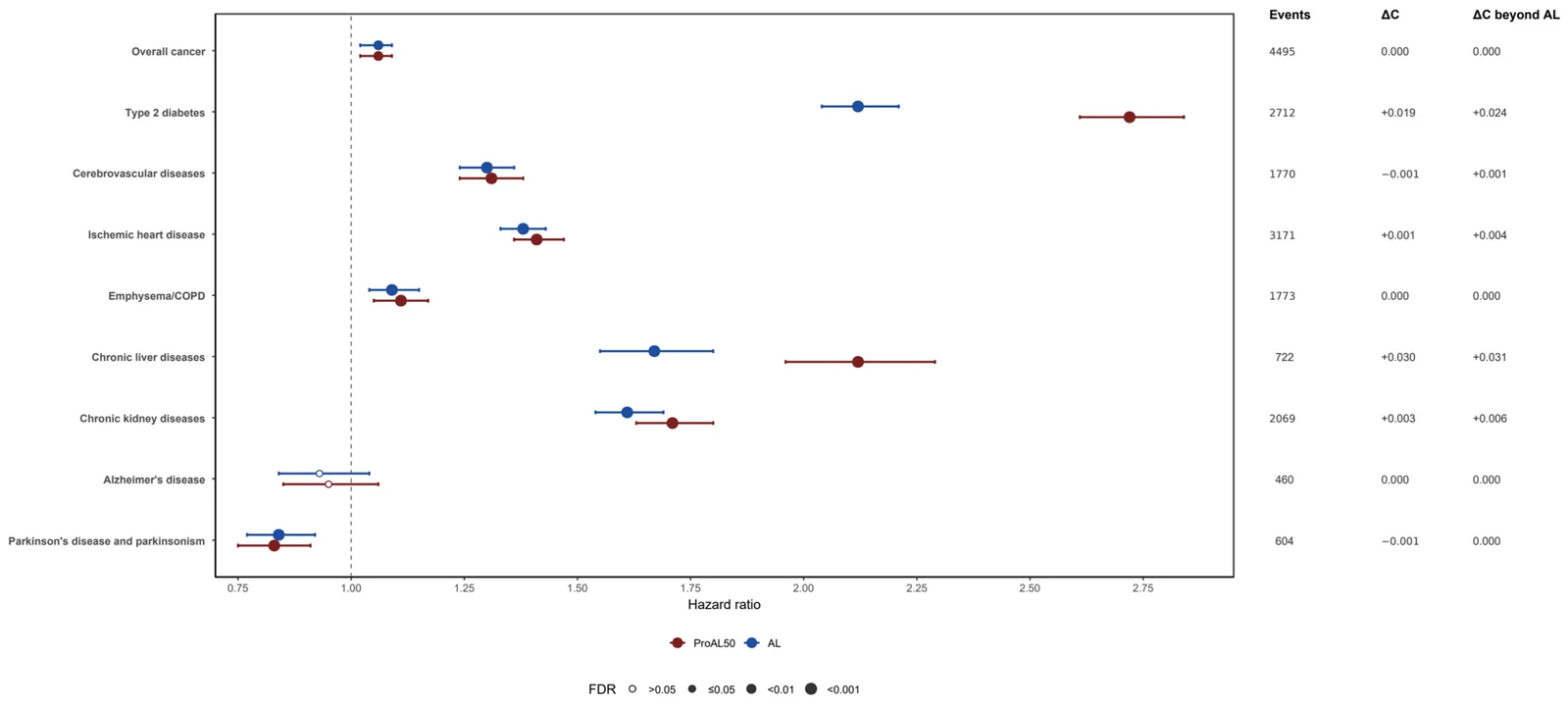
**Associations of allostatic load and ProAL50 with incident disease risk.** Hazard ratios from Cox proportional hazards models showing associations of AL and the ProAL50 proteomic score with incident chronic diseases and overall cancer in the UK Biobank. Models were adjusted for demographic, socioeconomic, lifestyle, and behavioral covariates. Points represent hazard ratios and horizontal bars indicate 95% confidence intervals; numbers of events are shown. Point color encodes exposure (blue = AL, red = ProAL50), and point size encodes FDR-adjusted significance level (<0.05, <0.01, <0.001); open points indicate non-significance (FDR > 0.05). ΔC represents the replacement contrast, C(Model 1 + ProAL50) − C(Model 1 + AL), whereas ΔC beyond AL represents the incremental contrast, C(Model 1 + AL + ProAL50) − C(Model 1 + AL). Bootstrap 95% confidence intervals for both ΔC measures are reported in Supplementary Table S5a.

Higher AL and ProAL50 were associated with the risk of multiple incident chronic diseases and overall cancer (Figure 4 and Supplementary Table S5). Overall, participants were followed for approximately 11.8 years, with outcome-specific median follow-up ranging from 11.70 to 11.86 years. Hazard ratios are presented per 1-SD increase in each marker. For overall cancer (Events = 4495), both AL and ProAL50 showed modest but significant adjusted associations (AL: HR = 1.06, 95% CI 1.02–1.09; ProAL50: HR = 1.06, 95% CI 1.02–1.09; ΔC = 0.000). In site-specific cancer analyses, associations varied by cancer type (Supplementary Table S6). Increased AL and ProAL50 were associated with elevated risks of kidney and cervical cancers. ProAL50 was additionally associated with higher risks of bowel and liver cancers, whereas the corresponding AL associations did not remain significant after FDR correction. For type 2 diabetes (Events = 2712), both AL and ProAL50 were significantly associated with incident disease, and replacing AL with ProAL50 in the fully adjusted model improved discrimination (AL: HR = 2.12, 95% CI 2.04–2.21; ProAL50: HR = 2.72, 95% CI 2.61–2.84; ΔC = 0.019). Similar patterns were observed for cerebrovascular disease (Events = 1770; AL: HR = 1.30, 95% CI 1.24–1.36; ProAL50: HR = 1.31, 95% CI 1.24–1.38; ΔC = −0.001), ischemic heart disease (Events = 3171; AL: HR = 1.38, 95% CI 1.33–1.43; ProAL50: HR = 1.41, 95% CI 1.36–1.47; ΔC = 0.001), emphysema/COPD (Events = 1773; AL: HR = 1.09, 95% CI 1.04–1.15; ProAL50: HR = 1.11, 95% CI 1.05–1.17; ΔC = 0.000), chronic liver disease (Events = 722; AL: HR = 1.67, 95% CI 1.55–1.80; ProAL50: HR = 2.12, 95% CI 1.96–2.29; ΔC = 0.030), and chronic kidney disease (Events = 2069; AL: HR = 1.61, 95% CI 1.54–1.69; ProAL50: HR = 1.71, 95% CI 1.63–1.80; ΔC = 0.003). In contrast, neither AL nor ProAL50 was significantly associated with Alzheimer’s disease after adjustment (Events = 460; AL: HR = 0.93, 95% CI 0.84–1.04; ProAL50: HR = 0.95, 95% CI 0.85–1.06; ΔC = 0.000). For Parkinson’s disease and parkinsonism (Events = 604), adjusted associations were inverse for both measures (AL: HR = 0.84, 95% CI 0.77–0.92; ProAL50: HR = 0.83, 95% CI 0.75–0.91; ΔC = −0.001). Overall, ProAL50 closely recapitulated the outcome associations of traditional AL, with the largest gains in comparative predictive discrimination when ProAL50 replaced AL observed for type 2 diabetes (ΔC = 0.019) and chronic liver disease (ΔC = 0.030). In mutually adjusted models containing both AL and ProAL50, ProAL50 remained associated with type 2 diabetes (HR per SD = 2.24, 95% CI 2.12–2.36), cerebrovascular disease (HR = 1.15, 95% CI 1.08–1.24), ischemic heart disease (HR = 1.23, 95% CI 1.17–1.30), chronic liver disease (HR = 2.00, 95% CI 1.80–2.22), and chronic kidney disease (HR = 1.42, 95% CI 1.33–1.52; all FDR < 0.05) after adjustment for traditional AL (Supplementary Table S5). Traditional AL also remained associated with type 2 diabetes, cerebrovascular disease, ischemic heart disease, and chronic kidney disease after adjustment for ProAL50. In contrast, mutually adjusted associations with overall cancer, emphysema/COPD, Alzheimer’s disease, and Parkinson’s disease/parkinsonism did not remain significant after FDR correction. When ProAL50 was added to the fully adjusted model already containing traditional AL, the largest incremental gains in discrimination were observed for chronic liver disease (ΔC = 0.031, 95% CI 0.020–0.041) and type 2 diabetes (ΔC = 0.024, 95% CI 0.020–0.028). In 2-year landmark sensitivity analyses, the associations with type 2 diabetes and chronic kidney disease remained materially unchanged. For type 2 diabetes, 36,009 participants remained at risk at the 2-year landmark, with 2405 subsequent events and a median post-landmark follow-up of 9.81 years (IQR 9.14–10.52); ProAL50 remained associated with incident disease in Model 1 (HR = 2.74, 95% CI 2.62–2.86) and in the mutually adjusted model (HR = 2.26, 95% CI 2.14–2.40), with an incremental ΔC beyond AL of 0.025. For chronic kidney disease, 36,899 participants remained at risk with 2003 subsequent events and a median post-landmark follow-up of 9.83 years (IQR 9.17–10.54); ProAL50 remained associated with incident disease in Model 1 (HR = 1.69, 95% CI 1.61–1.78) and in the mutually adjusted model (HR = 1.42, 95% CI 1.33–1.51), with an incremental ΔC beyond AL of 0.006 (Supplementary Table S5c). These findings reduce the likelihood that the primary associations were driven solely by diagnoses occurring shortly after baseline.

### 3.5. ProAL50 Predicts All-Cause and Cause-Specific Mortality

**Figure 5:**
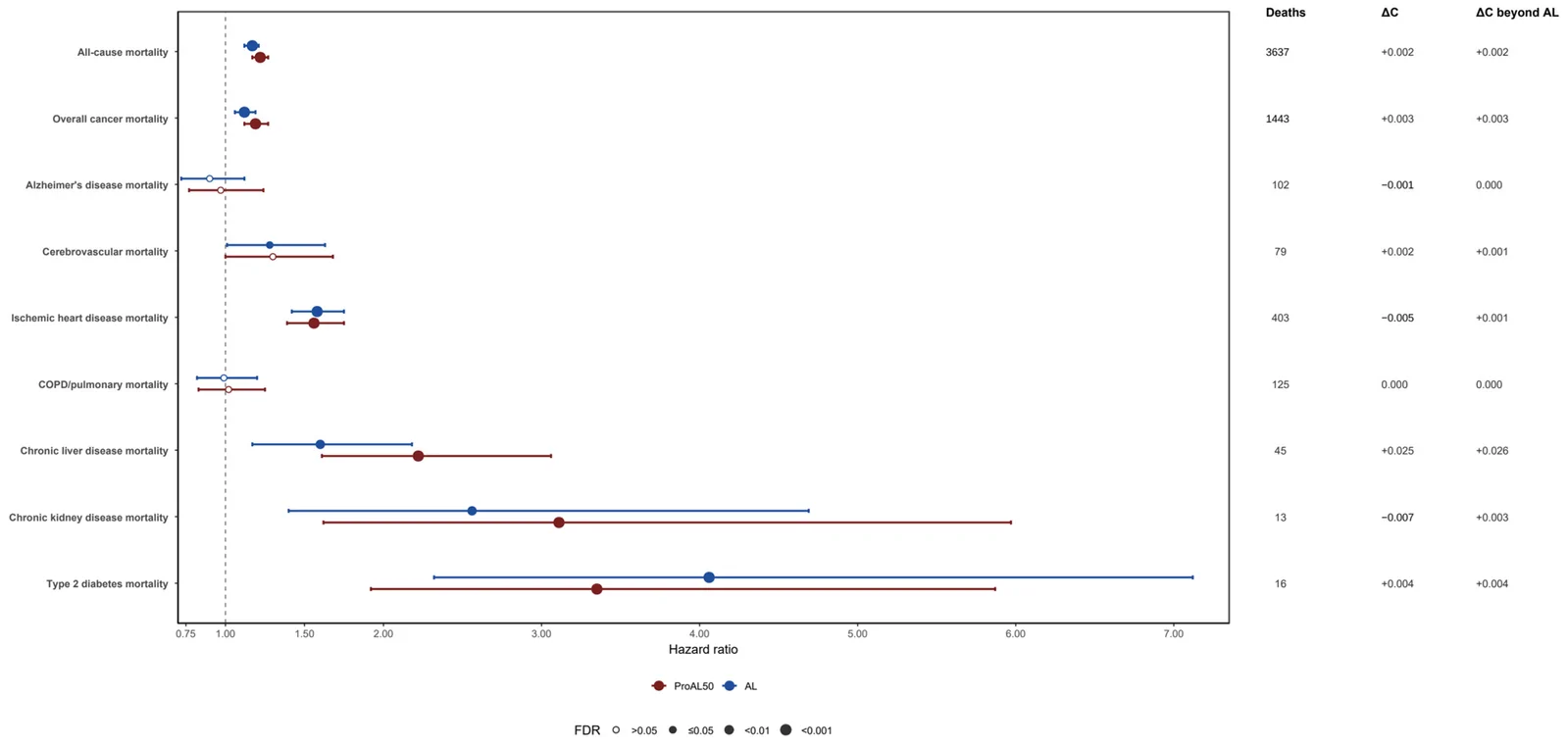
**Associations of allostatic load and ProAL50 with mortality outcomes.** Hazard ratios from Cox proportional hazards models showing associations of AL and the ProAL50 proteomic score with all-cause mortality and cause-specific mortality in the UK Biobank. Models were adjusted for demographic, socioeconomic, lifestyle, and behavioral covariates. Points represent hazard ratios and horizontal bars indicate 95% confidence intervals; numbers of deaths are shown. Point color encodes exposure (blue = AL, red = ProAL50), and point size encodes FDR-adjusted significance level (<0.05, <0.01, <0.001); open points indicate non-significance (FDR > 0.05). ΔC represents the replacement contrast, C(Model 1 + ProAL50) − C(Model 1 + AL), whereas ΔC beyond AL represents the incremental contrast, C(Model 1 + AL + ProAL50) − C(Model 1 + AL). Bootstrap 95% confidence intervals for both ΔC measures are reported in Supplementary Table S7a.

Higher AL and ProAL50 were associated with increased all-cause and cause-specific mortality, with associations that were directionally consistent between the two measures (Figure 5 and Supplementary Table S7). For all-cause mortality (Events = 3637), both measures remained significantly associated after adjustment, (AL: HR = 1.17, 95% CI 1.12–1.21; ProAL50: HR = 1.22, 95% CI 1.17–1.27; ΔC = 0.002). A similar pattern was observed for overall cancer mortality (Events = 1443; AL: HR = 1.12, 95% CI 1.06–1.19; ProAL50: HR = 1.19, 95% CI 1.12–1.27; ΔC = 0.003).

For non-cancer mortality outcomes, type 2 diabetes mortality showed large effect estimates despite few deaths (Events = 16; AL: HR = 4.06, 95% CI 2.32–7.12; ProAL50: HR = 3.35, 95% CI 1.92–5.87; ΔC = 0.004). Ischemic heart disease mortality was also significantly associated with both measures (Events = 403; AL: HR = 1.58, 95% CI 1.42–1.75; ProAL50: HR = 1.56, 95% CI 1.39–1.75; ΔC = −0.005). For cerebrovascular disease mortality (Events = 79), AL showed an FDR-significant association (HR = 1.28, 95% CI 1.01–1.63), whereas the ProAL50 association was nominally significant but did not remain FDR-significant (HR = 1.30, 95% CI 1.00–1.68). Both AL and ProAL50 were associated with chronic liver disease mortality (Events = 45; AL: HR = 1.60, 95% CI 1.17–2.18; ProAL50: HR = 2.22, 95% CI 1.61–3.06; ΔC = 0.025) and chronic kidney disease mortality (Events = 13; AL: HR = 2.56, 95% CI 1.40–4.69; ProAL50: HR = 3.11, 95% CI 1.62–5.97; ΔC = −0.007). In contrast, neither measure was significantly associated with emphysema/COPD mortality or Alzheimer’s disease mortality after adjustment.

In site-specific cancer mortality analyses, associations varied by cancer type and were generally stronger for ProAL50 (Supplementary Table S8). After adjustment and multiple-comparison correction, ProAL50 was significantly associated with mortality from breast, bowel, and liver cancer and non-Hodgkin lymphoma, whereas the association with kidney cancer mortality was nominal and did not remain FDR-significant. No FDR-significant adjusted associations were observed for AL across any site-specific cancer mortality outcome. Overall, ProAL50 showed mortality associations broadly comparable to traditional AL, although larger effect estimates were observed for ProAL50 for several site-specific cancer mortality outcomes, including liver cancer and non-Hodgkin lymphoma.

Mutually adjusted mortality analyses similarly showed evidence of both shared and partially independent associations. After simultaneous adjustment for traditional AL, ProAL50 remained associated with all-cause mortality (HR per SD = 1.17, 95% CI 1.11–1.23), overall cancer mortality (HR = 1.18, 95% CI 1.09–1.28), ischemic heart disease mortality (HR = 1.23, 95% CI 1.06–1.44), and chronic liver disease mortality (HR = 2.27, 95% CI 1.49–3.45; all FDR < 0.05). Traditional AL remained associated with all-cause mortality, type 2 diabetes mortality, and ischemic heart disease mortality after adjustment for ProAL50. Associations with cerebrovascular, COPD/pulmonary, chronic kidney disease, and Alzheimer’s disease mortality were attenuated after mutual adjustment (Supplementary Table S7). When ProAL50 was added to the fully adjusted model already containing traditional AL, the largest incremental gains in discrimination were observed for chronic liver disease mortality (ΔC = 0.026, 95% CI 0.006–0.050). Given the small number of deaths for several chronic diseases (e.g., type 2 diabetes and chronic kidney disease), the results for those diseases should be interpreted as exploratory.

### 3.6. Pathway Analysis of the ProAL50 Protein Panel

To characterize the biological functions represented by the 50-protein panel, we performed functional enrichment analysis using the STRING database. The queried annotation categories included Gene Ontology Biological Process, KEGG pathways, Reactome pathways, WikiPathways, UniProt Keywords, cellular-compartment annotations, and InterPro domains. STRING functional enrichment analysis identified eight FDR-significant terms, primarily reflecting extracellular localization, secretion, glycoprotein features, and growth factor-related functions (Supplementary Table S9). No Gene Ontology Biological Process, KEGG, Reactome, or WikiPathways term remained significant after FDR correction against the mapped Olink assay background. The eight surviving terms consisted of five UniProt Keywords, two cellular-compartment terms, and one InterPro domain. The most significantly enriched terms included Signal (43 proteins; FDR = 6.72 × 10^−6^), Extracellular region (34 proteins; FDR = 0.0015), Secreted (30 proteins; FDR = 0.0021), and Disulfide bond (37 proteins; FDR = 0.0021). Additional significant terms included Glycoprotein (37 proteins; FDR = 0.0045), Endoplasmic reticulum lumen (9 proteins; FDR = 0.0048), Growth factor receptor cysteine-rich domain superfamily (9 proteins; FDR = 0.0079), and Growth factor binding (4 proteins; FDR = 0.0119).

## 4. Discussion

In this study, we developed and validated ProAL50, a proteomics-derived index of AL, using high-dimensional plasma proteomic data from the UK Biobank with external construct validation in CARDIA. ProAL50 recapitulated many of the well-established sociodemographic, behavioral, physical-health, and mental-health correlates of traditional AL, supporting its construct validity as a proteomic marker of cumulative physiological dysregulation. In prospective analyses, ProAL50 was associated with incident chronic disease and mortality across multiple organ systems, providing predictive information comparable to, and for several outcomes exceeding, that of traditional AL. Together, these findings support ProAL50 as a biologically informative proteomic measure that complements traditional AL and may improve risk stratification in population-based research.

A central strength of ProAL50 is its strong construct validity, supported by consistent performance across internal validation, external validation, and multiple sensitivity analyses. Across sociodemographic characteristics, lifestyle factors, and general and mental health measures, ProAL50 showed patterns of association that closely mirrored those observed for traditional AL. Age, sex, socioeconomic position, neighborhood deprivation, smoking, physical activity, sleep disturbance, fatigue, psychological distress, and inflammatory burden were all consistently related to higher ProAL50 in directions and magnitudes comparable to AL. This concordance supports the interpretation of ProAL50 as a robust marker of cumulative physiological dysregulation rather than a disease-specific or pathway-restricted biomarker. The signature was also robust to key analytic choices: performance was stable in held-out internal validation and on external construct validation in CARDIA, the selected proteins were insensitive to the Elastic Net mixing parameter, panels of 20–200 proteins yielded highly correlated scores, and results were consistent across alternative continuous and three-level AL training targets. However, since ProAL50 was trained against an AL phenotype composed of conventional clinical biomarkers, it should therefore be viewed as complementary to, rather than a replacement for, traditional AL, and its biological coverage is influenced by both the AL training phenotype and the protein content of the Olink platform.

Beyond construct validity, ProAL50 provided predictive information for both disease incidence and mortality that was broadly comparable to traditional AL. For most outcomes, including overall cancer, metabolic disease, chronic lung disease, and chronic kidney disease, the two measures showed similar discrimination, with overlapping or only modest differences in C-statistics. In mutually adjusted models containing both measures, ProAL50 remained independently associated with several incident disease and mortality outcomes, whereas associations for other outcomes were attenuated, indicating both overlapping and partially independent outcome-related information. ProAL50 generally yielded only small incremental gains in discrimination beyond traditional AL. Thus, these findings indicate that ProAL50 performs comparably to traditional clinical AL for most outcomes while capturing additional dimensions of biological vulnerability for selected conditions.

The biological differences between these measures help explain both their broad concordance and the potential advantages of ProAL50. Traditional allostatic load (AL) indices are composites of static clinical measurements, many of which reflect downstream consequences of physiological dysregulation and are influenced by short-term physiological states, medication use, and measurement context [2,3,5]. In contrast, circulating proteomic profiles integrate genetic predisposition, cumulative environmental exposures, behavioral factors, immune activation, and metabolic regulation, providing a systems-level representation of physiological dysregulation [10,11,17,18,19,20]. Functional enrichment analysis further suggested that the proteins comprising ProAL50 are not a random subset of the Olink Explore platform. Using all mapped proteins measured by Olink Explore as the reference background, the ProAL50 panel remained significantly enriched for extracellular, secreted, glycoprotein, and growth factor-related proteins. However, no Gene Ontology Biological Process, KEGG, Reactome, or WikiPathways term remained significant after FDR correction. Because the operational AL index used to train ProAL50 did not include direct neuroendocrine measures and contained several lipid-related biomarkers, the enrichment analysis should not be interpreted as identifying novel biological pathways underlying allostatic load. Rather, it provides descriptive biological context for the localization and structural characteristics of the proteins selected into the ProAL50 panel.

From a translational perspective, ProAL50 overcomes several key limitations that have constrained the utility of traditional AL. ProAL50 should currently be considered a research-oriented proteomic surrogate rather than a validated targeted clinical assay. Traditional AL requires multiple heterogeneous measurements, fasting samples, and arbitrary cut-points and suffers from substantial missingness and poor cross-study comparability [3,5]. In contrast, ProAL50 can be derived from plasma proteomic measurements generated using a standardized research proteomic platform. The external construct validation in CARDIA, where ProAL50 correlated with an independently constructed AL score. supports its transportability, although the current weights are specific to the Olink platform, and prognostic validation in external cohorts remains needed. These features make ProAL50 particularly well suited for large biobanks, longitudinal cohort studies, and research settings where comprehensive clinical laboratory panels are unavailable or impractical.

Several findings also provide insight into the biological embedding of chronic stress. The strong associations of ProAL50 with physical functioning, fatigue, psychological distress, inflammatory markers, and accelerated biological aging suggest that proteomic AL may be especially sensitive to stress-related immune and metabolic dysregulation. The pronounced associations with kidney disease incidence and mortality, as well as liver disease mortality, are consistent with emerging evidence that chronic stress and systemic inflammation play central roles in organ vulnerability and failure [21,22,23]. For Parkinson’s disease and parkinsonism, both AL and ProAL50 showed positive crude associations but inverse associations after multivariable adjustment. These inverse estimates were attenuated in mutually adjusted models and may reflect reverse causation, residual confounding, or changes in health-related behaviors preceding diagnosis. These findings should therefore be interpreted cautiously. In contrast, neither measure showed a significant adjusted association with Alzheimer’s disease, which may also reflect the distinct pathobiology of neurodegenerative disorders and the potential importance of central nervous system–specific biomarkers [24,25]. We also observed a significant association between ProAL50 and ProAgeGap, a proteomic biomarker of biological aging [12]. This finding raises the question of the added value of ProAL50 given the growing number of biological aging biomarkers that have been developed. However, AL and biological aging represent related but conceptually distinct constructs. AL was developed to quantify the cumulative physiological consequences of chronic stress and repeated adaptive responses across multiple biological systems, whereas biological aging measures are generally designed to capture the rate or extent of age-related functional decline. Thus, although chronic stress may contribute to biological aging, AL is intended to capture stress-related physiological dysregulation rather than aging itself.

This study has several limitations. First, ProAL50 is a supervised signature anchored to traditional AL, so it inherits limitations of the AL construct itself, including a dichotomization-based training target and the use of standard rather than ancestry-specific thresholds; reassuringly, sensitivity analyses using a continuous AL target yielded broadly consistent results. The operational AL index used here did not include direct neuroendocrine mediators and included several lipid-related indicators; therefore, ProAL50 should be interpreted as a proteomic surrogate of this specific operationalization of AL. In addition, because hemoglobin A1c and creatinine contribute to the conventional AL index and are closely related to the clinical assessment of type 2 diabetes and chronic kidney disease, respectively, associations of ProAL50 with these outcomes may partly reflect information contained in the supervised training target. Importantly, the associations with type 2 diabetes and chronic kidney disease persisted in the 2-year landmark analyses, reducing the likelihood that these findings were driven solely by subclinical disease or near-term diagnoses at baseline. Second, although ProAL50 was externally validated, further validation in more diverse populations and clinical settings is warranted. The discordant association observed among Black UK Biobank participants should not be interpreted as evidence of a biological racial difference; the relatively small number of Black participants and predominantly White training population may limit transportability, highlighting the need for race-stratified validation in more diverse cohorts. In addition, although most UKB-PPP participants were broadly sampled, a subset was selected through consortium-specific disease-focused projects, resulting in enrichment for certain disease groups; this sampling feature should be considered when evaluating generalizability. Third, Olink NPX values are platform-specific relative abundance measures, and we did not perform cross-platform harmonization, batch-effect mitigation across independent proteomic platforms, or absolute protein quantification; additional work is therefore needed to evaluate assay portability, calibration, and reproducibility before ProAL50 can be implemented broadly across proteomic technologies or translated into clinical use. Fourth, while proteomic platforms are increasingly accessible, cost and infrastructure requirements may currently limit widespread implementation, though these barriers are rapidly diminishing. Fifth, although ProAL50 captures variation in circulating protein abundance, it does not distinguish individual proteoforms arising from alternative splicing, proteolytic processing, or post-translational modifications. These molecular variants may have distinct biological functions and could provide additional insight into stress-related physiology. Future studies integrating top-down mass spectrometry or other proteoform-resolved technologies may further refine proteomic signatures of allostatic load and identify biologically informative molecular variants beyond total protein abundance. Sixth, some cause-specific mortality and site-specific cancer analyses had limited event counts and should be interpreted cautiously. Finally, although we demonstrate strong associations with disease and mortality outcomes, causal inference remains limited; ProAL50 should be interpreted as a marker of vulnerability rather than a direct mechanistic mediator.

In summary, ProAL50 represents a proteomics-based surrogate of AL that preserves the conceptual foundation of traditional AL while providing a biologically richer, systems-level assessment of multisystem physiological dysregulation. ProAL50 mirrored established correlates of AL, showed external construct validity in CARDIA, and was associated with incident chronic disease and mortality that were broadly comparable to traditional AL, with selective added value for several outcomes. Rather than replacing traditional AL, ProAL50 may complement it in research settings where proteomic measurements are available but the individual clinical biomarkers required to construct AL are unavailable or inconsistently measured. These findings support proteomics-based AL measures as tools for population health research, chronic disease risk stratification, and studies of how chronic stress becomes biologically embedded to shape long-term health outcomes.

## Figures and Tables

**Table 1:** Sociodemographic and lifestyle determinants of AL and ProAL50 (N = 37,089).

		AL		ProAL50	
	N (%)	Coefficient (95%CI)	*p* Value	Coefficient (95%CI)	*p* Value
Age					
≤57	16,919 (45.62)	Ref		Ref	
>57	20,170 (54.38)	0.65 (0.61, 0.68)	<0.001	0.41 (0.39, 0.43)	<0.001
Gender					
Female	19,468 (52.49)	Ref		Ref	
Male	17,621 (47.51)	0.98 (0.95,1.01)	<0.001	0.83 (0.81, 0.85)	<0.001
Race					
White	34,671 (93.48)	Ref		Ref	
Black	758 (2.04)	0.09 (−0.03, 0.20)	0.15	−0.18 (−0.24, −0.11)	<0.001
Asian	850 (2.29)	0.23 (0.12, 0.34)	<0.001	0.18 (0.12, 0.24)	<0.001
Mix or others	688 (1.85)	−0.08 (−0.20, 0.04)	0.21	−0.07 (−0.14, −0.01)	0.032
Income					
≤30,999	15,473 (41.72)	Ref		Ref	
>30,999	16,116 (43.45)	−0.19 (−0.23, −0.15)	<0.001	−0.10 (−0.13, −0.08)	<0.001
Education					
High school or less	16,416 (44.26)	Ref		Ref	
College or professional	13,940 (37.59)	−0.22 (−0.26, −0.18)	<0.001	−0.11 (−0.13, −0.09)	<0.001
Townsend					
Low	18,213 (49.11)	Ref		Ref	
High	18,826 (50.76)	0.09 (0.05, 0.12)	<0.001	0.06 (0.04, 0.08)	<0.001
Smoke					
Never	20,158 (54.35)	Ref		Ref	
Previous	12,855 (34.66)	0.17 (0.14, 0.21)	<0.001	0.09 (0.08, 0.11)	<0.001
Current	3959 (10.67)	0.14 (0.09, 0.20)	<0.001	0.21 (0.19, 0.24)	<0.001
Alcohol					
Never	7461 (20.12)	Ref		Ref	
Moderate	13,568 (36.58)	−0.21 (−0.25, −0.16)	<0.001	−0.16 (−0.18, −0.13)	<0.001
Heavy	16,026 (43.21)	−0.27 (−0.31, −0.22)	<0.001	−0.24 (−0.26, −0.21)	<0.001
Physical activity					
Low	5851 (15.78)	Ref		Ref	
Moderate	12,032 (32.44)	−0.29 (−0.34, −0.24)	<0.001	−0.19 (−0.21, −0.16)	<0.001
High	12,115 (32.66)	−0.48 (−0.53, −0.43)	<0.001	−0.35 (−0.38, −0.33)	<0.001
Sleepless					
Never	8964 (24.17)	Ref		Ref	
Sometimes	17,624 (47.52)	0.10 (0.06, 0.14)	<0.001	0.05 (0.03, 0.08)	<0.001
Usually	10,467 (28.22)	0.22 (0.17, 0.26)	<0.001	0.13 (0.11, 0.16)	<0.001

## Data Availability

Data underlying this study were accessed through the UK Biobank under application number 94449. Researchers who meet UK Biobank eligibility criteria may obtain access to the same data by submitting an independent application via the UK Biobank Access Management System (https://www.ukbiobank.ac.uk/enable-your-research/apply-for-access, accessed on 24 September 2025). Code availability: Analysis scripts used for participant selection, data preparation, and statistical modeling may be shared by Prof. Hua Zhao (qqx7mw@virginia.edu) upon reasonable request. Release of code is contingent on approval from the relevant institutional and ethics committees, and approved requests will be processed within six months.

## Supplementary Materials

The following supporting information can be downloaded at: https://www.mdpi.com/article/10.3390/proteomes14030047/s1, Table S1: AL Cut off; Table S2: UKB disease codes; Table S3: Panel-size sensitivity analysis comparing ProAL20, ProAL50, ProAL100, and ProAL200; Table S4: Associations with General and Mental Health; Table S5: AL and ProAL50 in relation to incident overall cancer and chronic disease; Table S6: AL and ProAL50 in relation to site-specific incident cancers; Table S7: AL and ProAL50 in relation to all-cause and cause-specific mortality; Table S8: AL and ProAL50 in relation to site-specific cancer mortality; Table S9: STRING functional enrichment of ProAL50 using the Olink-assayed proteins as the reference background.
